# A Treatise on the Practice of Medicine

**Published:** 1847-10

**Authors:** 


ARTICLE IX.

*A Treatise on the Practice of Medicine.* By George B. Wood, M. D., Professor of Materia Medica and Pharmacy in the University of Pennsylvania; one of the physicians of the Pennsylvania Hospital; one of the authors of the Dispensatory of the United States of America, etc. In two vols. 8vo. Vol. 1 pp. 791. Vol. 2 pp. 840. Philadelphia: Grigg, Elliot, & Co. 1847. (From the publishers. For sale by A. H. & C. Burley, Chicago, Ill.)

The above work forms two handsome volumes neatly executed in all respects. After reading the title, we opened the volumes with an agreeable anticipation of finding within a really valuable work on the Practice of Medicine. To say that our anticipations were realized would give but a feeble impression of our estimation of the work. Our anticipations were founded upon our personal acquaintance with the author, and our knowledge of his ability, amply, to accomplish the task he had undertaken. During the several years that we were in attendance upon his lectures, in the University of Pennslvania, we also followed him through the wards of the Pennsylvania Hospital. During that period we listened with so much profit to his instructions, both didactic and clinical, that we formed the most exalted opinion of his acquirements as a therapeutist, both theoretical and practical, and also of his perfect command of language, and agreeable style of expression,—great and acknowledged essentials in an author, as well as a teacher. When we speak of our estimation of Dr. Wood's ability as a therapeutist we would not be understood to undervalue his acquintance with Nosology, Pathology, and the other departments of practical medicine, but merely to set forth his pre-eminence in this point, as giving to his work a *practical* value, which, without such capacity in its author, it could not possess. It is a fact which, in late years, has become notorious, that it is by no means the physician who makes with most ease a scientific diagnosis, and can give the most learned exposition of the Etiology, Classification, &c., of diseases, who is the most successful practitioner,—but that it is he who is most observant of the effects of remedies in a condition of health and disease, and who has perfectly at his command the shades of difference in his remedies, and aptness in applying them. But when we find these latter valuable traits combined, in the same mind, with habits of observation, and much knowledge by study and practice of the former also, we can wish for nothing more in an author of a work on practical medicine. Such a mind we confidently assert is possessed by Dr. Wood, and in proof of our assertion we will give our readers as a specimen of his style the entire article on "Pernicious Fever." This article we have selected, not as superior in style or critical analysis of the subject to others, but as affording to our readers, in this specimen of the work, a most excellent treatise on a variety of fever, seen in this city (Chicago,) but seldom, but more frequently in places somewhat further south than this point. It is however occasionally seen in almost all miasmatic regions, and is so seldom recognized, unless frequently occurring, and so uniformly fatal unless its peculiarities are detected and the proper treatment applied in time, that it will be as useful we hope to those who only occasionally meet with it, as to those who daily have it to treat.

Previous, however, to the insertion of this extract, it will be proper to give the reader an outline of the contents of the work, and some idea of the arrangement of the various subjects discussed.

Part I. treats of General Pathology, and Therapeutics. From the "prefatory remarks" to this part of the work will be gathered a sufficient insight into the manner in which the author disposes of this department of practical medicine.

"PREFATORY REMARKS.

"A Treatise on the Practice of Medicine, as the term is now generally understood, embraces all those branches of medical science, with the exception of Midwifery and Surgery, which have a direct reference to the knowledge and treatment of disease, and to the preservation of health.

"That department of the Practice which has for its object the *knowledge* of disease is called PATHOLOGY, and is divided into *general pathology* and *special pathology*, the former treating of what is common to diseases in general, or to a number of them, the latter of what is peculiar to individual diseases. The second great department of the Practice, or that which concerns the *treatment* of diseases, is called THERAPEUTICS, which may also be considered as *general* or *special*, according as it teaches the principles of treatment common to many complaints, or the particular course demanded by each one separately. The third department, which embraces the *means of preventing* disease, or in other words of preserving health, has received the name of HYGIENE.

"But, notwithstanding this scientific arrangement of the different subjects of practical medicine, it has been found that the natural mode of teaching is the most effective. A stronger impression is made on the mind of the student, and one more available for practical purposes, by presenting him with a vivid picture of each disease in all its bearings, as it must hereafter offer itself to his attention, than by distributing its dissected parts among the various sciences to which they respectively belong, and thus separating what he will afterwards be compelled to put together again in practice. The former plan, therefore, will be followed in the present work. But there are numerous points in relation both to pathology and therapeutics which are common to many diseases, and which may with great propriety be treated of in general, so as at once to render the subsequent study of particular diseases more easy, and to spare the necessity for much and wearisome repetition. Before proceeding, therefore, to an account of individual diseases, I propose to throw together, in the first part of the work, such principles and facts of universal or extensive applicability, as appear to me to be well established, avoiding speculation as much as possible, and seldom stopping to discuss the numerous hypotheses which have risen and disappeared, in rapid succession, in this as in other branches of medicine.

"*Definition of Disease.*—Disease may be defined to be a derangement of the organization, or of one or more of the functions of the body. But this definition is defective, like almost all others referring to natural processes, which so run into one another that a precise line of distinction can seldom be drawn between them. In the performance of every function, and in the condition of every organ, there is considerable diversity within the limits of health; and a state of things which, if continued, would constitute disease, may be so fugitive as not to merit the name; so that, both in point of degree and duration, it is often impossible to say, of any particular variation from the ordinary condition of the system, whether it is healthy or morbid. For practical purposes, however, perfect accuracy of distinction is unnecessary. Derangements have usually passed considerably beyond the boundary which separates health and disease, before they are brought to the notice of a physician.

"*Division of the subject.*—In this part of the work I shall treat, *first*, of the CONSTITUENT FORMS of disease, or of those derangements which, by their various combination, constitute diseases as we ordinarily see them; *secondly*, of the causes of disease considered generally, forming the subordinate branch of pathological science denominated ETIOLOGY; *thirdly*, of the exploration of disease, in other words, the modes in which diseases may be recognized, one disease distinguished from another, and the whole course of each traced to its probable termination—a branch of general pathology which may be designated as SYMPTOMATOLOGY; and *fourthly*, of the general principles of treatment, or GENERAL THERAPEUTICS. Whatever observations may be necessary on the subject of HYGIENE will be most conveniently made in connection with individual diseases."

It would require too much space to give our readers even a faint impression of the value of this part of the work; which comprises 219 pages; so that we must be content with the expression of our candid opinion that it is at once the most complete and concise treatise on General Pathology, which it has ever been our good fortune to peruse.

Part II. treats of Special Pathology and Therapeutics. We give the "prefatory remarks" to this part of the work, also, as comprising a more clear idea of the mode of treating the subject than we could express in the same number of words.

"PREFATORY REMARKS.

"The object of this part of the work is to treat of diseases individually; and each will be considered separately in all its relations.

"Diseases are certain associations or groups of morbid states or phenomena, offered by nature to the notice of the observer. If examined carefully, they will be found to consist of two different sets; one, embracing all the cases in which the true pathological condition gives its name to the disease, the other, those in which certain prominent effects or phenomena are considered as the disease; the real morbid condition being kept in the back ground. As examples of the former may be adduced pleurisy, pneumonia, and gastritis, or inflammation severally of the pleura, lungs, and stomach; of the latter, diarrhœa, dropsy, and the hemorrhages, in which the title is derived from the secretion or effusion, in other words, from a mere effect of the proper pathological condition, from which the secretion or effusion proceeds. Some may object to the propriety of admitting the latter group into the list of diseases; but, as it is upon the effects alluded to that the attention is chiefly fixed, while the true pathological state may not be obvious, and may even be a subject of dispute, it will be most convenient to comply with the general custom; especially as, to the uninstructed, these phenomena will always constitute the several diseases, and names expressive of them will always hold their place in the common language. To the physician, however, it is important to look beyond the phenomena to their causes, and to fix his attention upon these as the true objects of his concern.

"A vast amount of time and industry has been expended in the formation of systems of nosology. It is not the intention of the author to discuss their merits. Imperfect they all necessarily are; because diseases are not yet sufficiently understood to permit us to see clearly their mutual relations; and systems founded upon this basis must be constantly changing with new discoveries, and the adoption of new views. In this uncertainty, that plan of arrangement appears to the author to be the best, which is most convenient, and which may tend to direct the attention rather to what is positively known, than to the conjectures and peculiar opinions of authors. Such a plan is the one based upon the seat of the disease, and this it is here proposed to adopt.

"Diseases will be placed together, which are situated in the same parts; and no other attention, in the mere arrangement, will be paid to their mutual relations, than to form distinct groups, in each division, of such as may have the closest analogy. Upon comparing diseases, we find occasion to divide them into three great classes, having reference to their seat. The *first* class includes those diseases which occupy the whole system at the same time, and in which all the functions are simultaneously deranged. To the *second*, belong constitutional affections which may display themselves in local disease in any part of the system, but not in all parts at the same time. The *third* class embraces all the proper local diseases, or those which essentially affect some particular structure or function, and in which, any general phenomena that may be presented, are only secondary. This portion of the work will, accordingly, be distributed into three divisions, corresponding with the classes mentioned."

"GENERAL DISEASES.

"The only diseases which belong strictly to this division are fevers, No other acute affections involve, like these, all the functions of the body; and if, in certain chronic affections, the system may become in some instances universally diseased, it is only in the advanced stages; and this universality is not, as in fevers, an essential part of their constitution.

"It will be recollected that, in the essay upon fever, in the first part of this work, the distinction between the essential idiopathic fevers, and the symptomatic were fully recognized. It is only of the former that I propose to treat in this place. Symptomatic fever is dependent solely on the local affection, owes all its importance to that affection, and ceases along with it. In fact it is the inflammation that constitutes the disease. The phlegmasiæ, therefore, as those diseases are called which consist of inflammation and consequent fever, are ranked along with the diseases of the organs in which they are severally seated.

"Idiopathic fevers have been variously divided and subdivided, and have received a great diversity of names, according to the views of different writers. Thus we have *intermittent*, *remittent*, and *continued* fevers; *synocha*, or *inflammatory fever; typhous*, *asthenic* or *adynamic fever;* and *synochous* or mixed fever, beginning as *synocha* and ending as *typhous*. To these, *congestive fever* has been recently added. But these diversities have reference merely to difference of form, grade, or type, and any one fever, that is, any febrile disease distinguised from others by the nature of its cause, may have all these different characters. Thus, the same fever, produced by the same cause, may be in different individuals, or in the same individual under different circumstances, either intermittent, remittent or continued, inflammatory, typhous, synochous, or congestive. Again, many distinct fevers have been made out of accidental complications; such as *gastric*, *gastro-enteritic*, *hepatic*, and *cerebral* fevers, so called in consequence of the predominance of disease in the several organs which gave origin to the names. But this nomenclature, so far as it has been applied to idiopathic fevers is incorrect; as it would seem to imply some essential difference between the diseases thus distinguished, whereas they may be absolutely the same disease, merely diversified by the occurrence of inflammation or irritation in one organ rather than in another. But, in very many instances, the diseases named as above have been nothing more than cases of phlegmasiæ, and would have been more properly entitled gastritis, gastro-enteritis, hepatitis, and encephalitis. It appears to me that, in the arrangement of fevers, we should endeavor to find out some essential difference between them, something which characterizes them as distinct diseases, peculiar in their phenomena and nature, and incapable of being converted into each other. Now such a basis of arrangement is offered in the peculiarity of the cause. Upon examining the various fevers considered essential or idiopathic, we find that, as a general rule, certain individuals are produced by one cause, others by another, others again by a third, and so on through almost the whole list; and we find further, that those produced by one of these causes cannot be produced by another, each set requiring its own particular cause. Here, then, is an excellent, and, as it appears to me, quite unobjectionable ground of association. The cases produced by the same cause may very properly be treated as belonging to the same disease; and any incidental peculiarities of form, type, &c., should serve only as the ground of varieties.

These different diseases have only one thing in common; namely, that all are attended with that proximate constituent of disease, called abstractly fever, or febrile movement. There is only one among them, in the arrangement of which, upon this plan, any great difficulty exists; and the difficulty, in this instance, arises from our ignorance of the cause. Nevertheless, in relation to that particular affection, there is so much to identify it in its phenomena, course, circumstances of occurrence, and anatomical characters, that we can scarcely deny its claim to individuality, though its cause is hidden, or at best obscure. The disease alluded to is that denominated variously, *continued* fever, *nervous* fever, *slow or protracted remittent*, *typhoid* fever, *typhus mitior*, *dothinenteritis*, *&c.*, and which I propose to denominate *enteric fever*.

"Proceeding upon the plan above laid down, I shall treat of the following fevers, as distinct diseases, viz:—

| | |
|---|---|
| 1. Irritative fever, [fever, | 7. Small-pox, or Variola, |
| 2. Miasmatic, or bilious | 8. Vaccine disease, or vaccina, |
| 3. Yellow fever, [fever, | 9. Chicken-pox, or varicella, |
| 4. Enteric, or typhoid | 10. Measles, or rubeola, |
| 5. Typhus fever, | 11. Scarlet fever, or scarlatina, |
| 6. Plague, | 12. Erysipelas. |

"Of several of these there are varieties which require distinct notice, and some of them distinct designations. Thus, miasmatic or bilious fever is distinguished into intermittent, and remittent, each of which may be mild or malignant, or to use a phraseology now fashiohable, inflammatory or congestive. Of the fevers above mentioned some have circumstances in common which might serve to associate them in distinct subdivisions. Thus, some are propagated by contagion, and are hence called *contagious fevers*; some have the property, in common, that they are attended with an eruptive affection, and are denominated *eruptive* or *exanthematous fevers*; Now it happens that these are in many instances interchangeable terms; most contagious fevers being exanthematous, und most of the exanthemata contagious. But this is not universally the case. The contagious and exanthematous fevers are entirely distinct individually; for, though they have certain points of similarity, each has its own peculiar traits, and its own peculiar cause."

"CONSTITUTIONAL DISEASES, &C.

"The above title does not exactly designate the diseases belonging to this class. It is used for the sake of brevity, with a somewhat arbitrary application. As already stated (*see page* 221), the second class embraces constitutional affections, which may display themselves in local disease of any part of the system, but not in all parts at the same time. This want of universality excludes them from the first class: and, as they frequently occupy several different organs at once, and may pass from any one organ to any other during the same attack, they cannot be placed in the category of affections strictly local.

"The only diseases which I place in this class are rheumatism and gout. There are others that properly belong to it, but, for convenience sake, are considered elsewhere. One of these is scrofulous or tuberculous disease. This is certainly a constitutional affection, and may show itself in any one part, or in many parts of the system at the same time. But the local affections are of so fixed a character, are in some instances so strongly marked, and so universally looked upon as constituting distinct diseases, that they are advantageously described rather in reference to their position than their nature. Hence, I have treated of what concerns the disease generally under general pathology, and propose to treat of its local exhibitions, as phthisis, external scrofula, mesenteric disease, &c., among the local affections belonging to the third class. The same remarks are applicable to carcinoma or cancer, and to the melanosis. Spyhilis, in its advanced stages, would also be attached to the present class, were it admitted into this work; but it is so generally considered as a surgical disease, and so fully treated of by surgical writers, that it may be omitted without inconvenience, in a treatise upon the practice of medicine. With regard to rheumatism and gout, it may be thought that the fever which attends them should rank them in the first class; but fever is not a necessary accompaniment of these diseases; and when it occurs, is almost always secondary, and dependent on the local affection.

"LOCAL DISEASES.

"This is much the largest of the three classes in which diseases are arranged in the present treatise. It embraces all those which have their seat primarily or essentially in any one organ, tissue, or function. The local affection is often accompanied with constitutional symptoms; but these are secondary: as in the phlegmasiæ, in which the fever depends on the inflammation. It is true that, among the following diseases, are also many which are results of constitutional derangement; but, in these instances, the local affection is so striking and important as chiefly to engage attention, and always to have ranked among diseases, with a distinct title; while the constitutional disorder, from which it may have sprung, is often concealed and unknown. Such are the local tuberculous affections, many instances of dropsy, and not a few cutaneous eruptions.

"I have preferred the several functions as the basis of arrangement in this class, in the region of the body; because it often happens, that a particular disease, though confined to one function, overleaps the region, and may in fact occupy several regions, as dropsy occuring at the same time in the chest, abdomen, and external areolar tissue. In the order of the functions, I begin with that which nature has placed first and lowest in the scale, and follow her course through the remainder. Pursuant to this plan, the diseases conected with the digestive system come first in order; then those of the absorbent system; and afterwards successively those of the respiratory, circulatory, secretory, nervous, and reproductive systems.

"In each group of diseases, those which consist in inflammation of the parts concerned are first treated of; because they are in general better understood and more easily recognised, and consequently, when known, serve as standards of comparison for the more obscure functional affections."

The article on Pernicious Fever, which we promised our readers in our opening remarks, for want of room we are obliged to omit until our next number. We shall accordingly close our notice of the admirable work whose title leads our article with the single remark, that we recommend it with all confidence as the most complete and perfect work on the practice of medicine which has ever been issued from the American press. J. V. Z. B.

